# *MLH1* Ile219Val Polymorphism in Argentinean Families with Suspected Lynch Syndrome

**DOI:** 10.3389/fonc.2016.00189

**Published:** 2016-08-24

**Authors:** Mev Dominguez-Valentin, Patrik Wernhoff, Andrea R. Cajal, Pablo G. Kalfayan, Tamara A. Piñero, Maria L. Gonzalez, Alejandra Ferro, Ines Sammartino, Natalia S. Causada Calo, Carlos A. Vaccaro

**Affiliations:** ^1^Department of Tumor Biology, Institute for Cancer Research, Oslo University Hospital, Oslo, Norway; ^2^Unit of Muscle Biology, Lund Transgenic Core Facility/Reproductive Immunology, Department of Experimental Medical Science, Lund University, Lund, Sweden; ^3^Institute of Basic Sciences and Experimental Medicine (ICBME), Instituto Universitario Hospital Italiano, Buenos Aires, Argentina; ^4^Programa de Cancer Hereditario (ProCanHe), Hospital Italiano de Buenos Aires, Buenos Aires, Argentina

**Keywords:** Ile219Val, Lynch syndrome, *MLH1*, *MSH2*, mutation

## Introduction

Heredity is a major risk factor for colorectal cancer (CRC). Identification of individuals and families at increased risk allows for targeted surveillance, which has been shown to reduce morbidity and mortality from CRC (1, 2).

Lynch syndrome is a multi-tumor syndrome with particularly high risks for colorectal, endometrial, and ovarian cancer (3–6). The syndrome is caused by germline DNA-mismatch repair (MMR) gene mutations with major contributions from *MLH1* (MIM#120436) (42%), *MSH2* (MIM#609309) (33%), *MSH6* (MIM#600678) (18%), and *PMS2* (MIM#600259) (8%). Only about one-third of the Lynch syndrome families fulfill the Amsterdam criteria (AC) (7–9). The cumulative incidence of any cancer at 70 years of age is 72% for *MLH1* and *MSH2* mutation carriers but lower in *MSH6* (52%) and *PMS2* (18%) mutation carriers. *MSH6* and *PMS2* carriers developed no cancers before 40 years of age (10).

Mutation screening in a relatively large proportion of South American families with suspected Lynch syndrome has recently identified 99 disease-predisposing mutations in *MLH1* and *MSH2*, which mutation spectrum is predominated by *MLH1* (60%) and *MSH2* (40%). Among the reported mutations, genetic hot-spot regions, new and potential founder mutations have been described in the South American population (11, 12).

Several genome-wide association studies have identified single nucleotide polymorphisms (SNPs) in at least 15 independent loci associated with CRC risk (odds ratio ranging from 1.10 to 1.26 per risk allele) (13–15). Although there is no evidence that these SNPs associated with CRC in the general population are modifiers of the risk for MMR gene mutation carriers overall and therefore any evidence of proven clinical utility in Lynch syndrome (16).

The *MLH1* Ile219Val (rs1799977) is a common germline alteration, located in exon 8 at the nucleotide 655. This polymorphism has been described in a high frequency of the South American Lynch syndrome population, but no modifier effect of CRC risk and MMR disease-predisposing mutation carriers was observed (17). However, it has been reported to confer a twofold-increased risk of CRC development in sporadic Mexican patients (18). Other conditions that have been associated with this polymorphism include childhood acute lymphoblastic leukemia, breast cancer, radiation-induced rectal or bladder toxicity, and ulcerative colitis (19–23). It is unknown whether the *MLH1* Ile219Val polymorphism has an effect on cancer risk and in the MMR capacity in Argentinean families with suspected Lynch syndrome. Thus, we aim to determine its frequency, its correlation with disease-predisposing MMR gene mutations, and to delineate the clinical characteristics from these families.

## Patients and Methods

### Ethics Statement

The study was performed in compliance with the Helsinki Declaration and approved by the Ethics Committee of the Hospital Italiano de Buenos Aires in Argentina. Written informed consent was obtained from all participants during genetic counseling sessions.

### Patient Selection

The Hereditary cancer registry of the Hospital Italiano de Buenos Aires is a national Argentinean registry that contains information of all families identified with proven or suspected hereditary cancer. Through research collaborations, data from the registry is freely available. This prospective database contains clinical, molecular, and familial data, which is clinically relevant. In addition, the electronic medical records of these patients can be reviewed to retrieve further data.

We obtained molecular (genetic testing) and epidemiological data (family history) from 48 families that fulfilled AC (*n* = 33), Bethesda guidelines criteria (*n* = 8), and families suggestive of a dominant CRC inheritance syndrome (*n* = 7) (7, 8, 24) between 2009 and 2016. Family pedigree was constructed based on the information provided by the proband during one or more genetic counseling sessions.

### Disease-Predisposing MMR Gene Mutations and Nomenclature

Germline mutation screening of *MLH1, MSH2, MSH6*, and *PMS2* was performed by next generation sequencing platform using the personal genome machine^®^ system (PGM, LT) according to the manufacturer’s instructions. Mutation nomenclature followed the Human Genome Variation Society (HGVS) guidelines.^1^ All identified mutations were compared to previously reported in MMR databases, maintained by the International Society for Gastrointestinal Hereditary Tumors^2^ and the French MMR network.^3^

### Statistical Analysis

Age comparison between the groups was performed using Mann–Whitney *U*-test. In the tables, results were expressed, as mean age ± SD. Significance for expected frequencies was tested using Chi-squared test. The analyses were performed using Statview (SAS Institute Inc. 100 SAS Campus Drive Cary, NC 27513-2414, USA) and Chi-squared Calculator (Social Science Statistics^4^). For all tests, *p* < 0.05 was considered statistically significant.

## Results

Overall, forty-eight hereditary CRC families were identified from the Hereditary cancer register of the Hospital Italiano de Buenos Aires. Of them, 19/48 (40%) of the families carried disease-predisposing mutations in the MMR genes. The mean age at first diagnosis in Lynch syndrome families was 42 ± 7.3 years (range 27–55). Mutations in *MLH1* and *MSH2* genes were identified in 17/33 (52%) of the families that fulfilled the AC. However, no disease-predisposing mutations in *MSH6* and *PMS2* genes were found in this series (Table 1). Mutational and clinical information from the Argentinean Lynch syndrome families is described in Table 1.

**Table 1:** **Disease-predisposing MMR gene mutations in Argentinean Lynch syndrome families**.

Family ID	Gene	Nucleotide	Consequence	Exon	Reported as	Clinical criteria	Cancer (age)
52	*MLH1*	c.199 G > A	p.G67R	2	Causal	ACI	No cancers
6	*MLH1^a^*	c.676 C > T	p.R226X	8	Causal	ACI	CRC (36)
8	*MLH1*	c.677 G > A	p.R226Q	8	Causal	ACI	CRC (38), EC (52)
DE	*MLH1*	c.677 + 5 G > A		8i	Likely causal	ACI	CRC (27), BC (29)
B	*MLH1^a^*	c.1852_1854 delAAG	p.K618del	16	Causal	ACI	EC (39), CRC (47)
50	*MLH1^a^*	c.1852_1854 delAAG	p.K618del	16	Causal	Bethesda	CRC (44)
2	*MLH1*	c.1890Dup	p.D631fsX1	16	Causal	ACI	CRC (50)
P	*MLH1^a^*	c.2252_2253delAA	p.K751SfsX3	19	Causal	ACI	EC (52)
DO	*MSH2*	c(?_−0.68)_211+?del		1	Causal	ACI	CRC (55)
51	*MSH2^a^*	c(?_−0.68)_211+?del		1	Causal	ACI	CRC (37), EC (46)
4	*MSH2*	c.166 G > T	p.E56X	1	Causal	ACI	CRC (44), EC (51)
3	*MSH2*	c.289 C > T	p.Q97X	2	Causal	ACI	CRC (49), SKC (51), UT (52)
SS	*MSH2*	c.484 G > A	p.G162Arg	2	Causal	ACII	No cancers
49	*MSH2*	c.388_389delCA	p.Q130ValfsX2	3	Causal	ACI	CRC (51), SKC (48), SIC (65)
DA	*MSH2*	c.1046C > G	p.P349R	6	Causal	ACI	BC (41)
L	*MSH2*	c.1662-2A > G		10i	Likely causal	Bethesda	CRC (42)
N	*MSH2*	c.1861C > T	p.R621X	11	Causal	ACII	EC (34), CRC (59), SIC (79)
45	*MSH2*	c.1910delC	p.R638GfsX47	12	Causal	ACII	CRC (39), EC (41), SIC (44), BC (49)
5	*MSH2*	c.2046_2047 delTG	p.V684DfsX14	13	Causal	ACII	CRC (42)

*^a^MLH1 I219V polymorphism*.

*CRC, colorectal cancer; EC, endometrial cancer; BC, breast cancer; SKC, skin cancer; UT urinary tract career; SIC, small intestine cancer; AC, Amsterdam criteria*.

When we analyzed the genotypic and allelic frequencies of the *MLH1* Ile219Val polymorphism, we found *Val*-carriers in 44% (21/48) of the Argentinean families with suspected of Lynch syndrome. Of them, 95% (20/21) were *Ile/Val* heterozygotes and 5% (1/21) were *Val/Val* homozygotes. The allelic frequency of *Ile* and *Val* were 0.77 and 0.23, respectively (Table 2).

**Table 2:** **Genotypic and allelic frequency of the *MLH1* I219V polymorhism in Argentinean families with suspected Lynch syndrome**.

Polymorphism	Genotype	*N*+/*N* total	Genotypic frequency	Allelic frequency
	Ile/Ile	27/48	0.56	
I219V	Ile/Val	20/48	0.42	0.77
C.655 A > G	Val/Val	1/48	0.02	0.23

*Ile/Ile: homozygosis for the wild allele; Ile/Val: heterozygosis; Val/Val: homozygosis for the polymorphic allele; N+: positive cases for each genotype; N total: total number of the evaluated individuals*.

Regarding the clinical characteristics, a slightly early mean age at CRC and endometrial cancer diagnoses was observed in the families without the *MLH1* Ile219Val polymorphism in comparison to the families harboring the *MLH1* polymorphism (45 and 44 years vs. 49 and 46 years, respectively) (*p* > 0.05). The presence of AC and Bethesda guidelines was not associated with the *MLH1* Ile219Val polymorphism (*p* > 0.05). As expected, CRC and endometrial cancer were the most frequent tumors among the *MLH1* Ile219Val carriers and non-carriers. While skin tumors were observed in both groups of families, breast and small intestine tumors were observed only in the non-*MLH1* Ile219Val families.

Of the 21 families with the *MLH1* Ile219Val polymorphism, 5 (24%) harbored disease-predisposing MMR gene mutations; *MLH1* was the most affected gene occurring in 4/5 (80%) of the cases. In the 27 non-*MLH1* Ile219Val polymorphism families, 14 (52%) harbored *MLH1* or *MSH2* disease predisposing mutations; *MHS2* the most frequently affected gene occurring in 10 of them (71%) (available upon request). In the group of *MLH1* Ile219Val polymorphism (*n* = 21) that displayed disease-predisposing MMR gene mutations (*n* = 5/21), the *Val*-allele was significantly associated with *MLH1* and/or *MSH2* mutations (*p* < 0.05).

In addition, we analyzed the clinical features of the 21 families harboring *MLH1* Ile219Val polymorphism in the presence of disease-predisposing MMR gene mutations, MMR carrier’s families showed an early mean age at CRC diagnosis (41 ± 5.35) years, although these differences were not significant (*p* > 0.05) (available upon request).

In order to evaluate if the *MLH1* Ile219Val polymorphism exerts any influence at the age of CRC onset, we analyzed the mean age at CRC diagnosis of families harboring or not the *MLH1* Ile219Val polymorphism according to their MMR status (*MLH1* or *MSH2*). The *MLH1* Ile219Val polymorphism group (*n* = 21), which harbored disease-predisposing mutations in *MLH1*, or *MSH2* showed a mean age at CRC onset of 42.3 and 37 years, respectively. In the non-*MLH1* Ile219Val carriers, the mean age among those who displayed the mutations was 38.3 and 47.6 years, respectively. However, no statistically significant association was found between these groups (available upon request).

## Discussion

In the Argentinean suspected Lynch syndrome families, the genetic polymorphism (Ile219Val) in the *MLH1* gene was found in 44% of the population, which fits with the previous genetic studies that reported an incidence of 31–80% from different populations (17, 24–26). The allelic frequencies for *Ile* and *Val* (0.77 and 0.23) were similar to the reports from South American suspected Lynch syndrome families (0.7 and 0.3), German Lynch syndrome families (0.69 and 0.31), Swedish Lynch syndrome families (0.64 and 0.34), and Italian suspected Lynch syndrome families (0.33 for *Val*) (17, 24–27). This similarity may be explained by the influence of European ancestors in the South American population, particularly in Brazil, Uruguay, and Argentina (11). However, our allelic frequencies are slightly higher than the minor allele frequency (MAF) reported by the 1000 Genomes Project (0.87 and 0.13, respectively) although similar to Puerto Rico when analyzed into the American population (28).

In the current study, no association was observed between the *MLH1* Ile219Val polymorphism and cancer risk. This is consistent with several functional analyses indicating that the variant has binding properties to *PMS2* and DNA repair efficiency similar to the wild type (29, 30). A recent CRC meta-analysis including 8068 cases and 6568 controls from Australia, Czech Republic, Spain, Germany, and Sweden with frequencies of 0.678 and 0.671, respectively, found not associations of CRC risk and *MLH1* Ile219Val polymorphism (31). However, Kim et al. suggested that the homozygosity for the 219V variant was correlated with a significantly reduced *MLH1* expression among sporadic CRC cases (32). The difference might be a reflection of environmental impact on gene distribution, ethnic background or small-sized family cohort (33–35).

We found that the absence of the *MLH1* Ile219Val polymorphism was associated with an early mean age at CRC cancer diagnosis, and the development of breast tumors was the main clinical features. This is in line with the Brazilian hereditary non-polyposis colorectal cancer (HNPCC) familial study that reported that breast cancer followed by endometrial and uterine cervix cancer are the most frequent extracolonic tumors found in women (36). Although, this is a small increase in breast cancer risk, it might point out interesting biological connections.

Regarding the association of the *MLH1* Ile219Val polymorphism and disease-predisposing MMR gene mutations, we described a lower frequency (24%) of disease-predisposing MMR gene mutations compared to the reported by the South American Lynch syndrome families (33%) (17). In families harboring the *MLH1* Ile219Val polymorphism and disease-predisposing MMR mutations, *MLH1* was the most affected gene. The seemingly unique contribution than the 57 (*MLH1*) and 43% (*MSH2*) reported by the South American population could reflect random variation, population structure, size sample and genetic heterogeneity (17). In this series, we found a statistically significant link between the presence of the *MLH1* Ile219Val polymorphism and disease-predisposing MMR gene mutations carriers, which suggest that these genetic discriminators may be relevant for molecular diagnostics in this population. However, our study has a limited sample size and the results need further validation. Studies with larger sample size are needed to further evaluate the role of this polymorphism.

In summary, our findings point to a high frequency of the *MLH1* Ile219Val polymorphism in the Argentinean families with suspected Lynch syndrome and its modifier effect with the disease-predisposing *MLH1/MSH2* genes mutations. Our data may provide important clues to contribute to molecular diagnostics, improved risk stratification, and targeted therapeutic strategies in hereditary CRC.

## Author Contributions

MD-V, PW, AC, PK, AF, MG, IS, NC, TP, and CV participated in the acquisition of data, or analysis, interpretation of data and have been involved in drafting the manuscript. All authors read and approved the final manuscript.

## Conflict of Interest Statement

The authors declare that the research was conducted in the absence of any commercial or financial relationships that could be construed as a potential conflict of interest.

## Footnotes

^1^www.hgvs.org/mutnomen/

^2^http://www.insight-group.org

^3^http://www.umd.be/UMD_Central/

^4^http://www.socscistatistics.com
